# Undermine Sufferers’ Testimonies to Avoid Social Impacts of Pain

**DOI:** 10.3390/healthcare11091339

**Published:** 2023-05-06

**Authors:** Mª Isabel García-Rodríguez, Lourdes Biedma-Velázquez, Rafael Serrano-del-Rosal

**Affiliations:** Institute for Advanced Social Studies, Spanish National Research Council (IESA-CSIC), 14004 Córdoba, Spain

**Keywords:** pain, epistemic injustice, social order, public health

## Abstract

Pain is a subjective experience that is mediated by the social structure and by the contextual aspects of people in pain. From the point of view of those affected, a sociological analysis has been carried out of why society doubts pain and the impact that the lack of credibility has on people in pain. Qualitative methodology is used. In total, 19 semi-structured interviews have been conducted with men and women in pain. Research has shown that pain produces discredit in all dimensions of individual’s social life, from the most intimate to that related to healthcare and production. The lack of credibility takes the form of epistemic injustice, being a reaction produced from the social structure to avoid the impacts that pain could produce on the social system. Epistemic injustice affects anyone in pain, but the form it takes will be related to sufferer’s circumstances. Studying this topic is important because it shows the rigidity of expert systems to deal with some old and new situations related to pain. It also shows the frequent lack of fit between the systems and the sufferers. Finally, the article shows that to deal unfairly with the testimony of people in pain has negative consequences on the treatment of pain. A better understanding of these issues could improve the sufferers’ living conditions.

## 1. Introduction

The research on pain is a recent topic in the field of sociology. “Although chronic pain (often abbreviated below as pain for parsimony) has been studied extensively in the biomedical and psychological sciences, sociological research of pain -despite important analytic contributions- has not yet coalesced into an unified subfield” [1]. Pain is a complex phenomenon with physiological, sensorial, affective, and cognitive dimensions [2].

Conceptualization of pain has evolved throughout history [3]. Pain is currently defined by the International Association for the Study of Pain (hereinafter IASP) as “an unpleasant sensory and emotional experience associated with, or resembling that associated with actual or potential tissue damage” [4].

Its difficulty to be objectified is such that some specialists have summed it up by saying that pain is anything that a patient says that hurts [5]. In the last decades, the analysis and conceptualization of pain suffered a paradigm shift, going from a strictly biomedical model, which gives priority to physiological issues, to a biopsychosocial model, also paying attention to pain’s psychological and social aspects. Honkasalo [6] analyzed how social practices have an influence on the configuration of pain and explained how sufferers bestow meaning in their daily life [7].

On his part, Zajacova et al. [1] exposed the pain interpretation frameworks up to the present. This is currently a phenomenon that has social and health effects. This is also unevenly distributed among the population, so this could be considered a population wellbeing barometer. In fact, numerous studies show that increased morbidity in chronic pain, particularly among the midlife population in the US, is related to different aspects of their living conditions [8].

Pain has clear social determinants [9]. Through the social practices of sufferers, pain establishes a dialectic relation with the social structures making up social order. By pressuring and destabilizing social structures, pain reveals that their structures have a limited ability to absorb pain-related events. Consequently, a conservative reaction takes place, aimed at maintaining the status quo through different strategies, namely, social control, delegitimization of pain and undermining sufferers’ credibility. The latter takes place with the production and maintenance of epistemic injustices.

This article analyzes how the forms of injustice, that are purely epistemic, are produced, that is, those that wrong someone in their capacity as a knower [10,11]. Fricker [11] distinguishes two main forms: testimonial injustice, which occurs when “prejudices lead a listener to accord a speaker’s words a diminished degree of credibility”, and hermeneutic injustice, which takes place when a gap in the resources of collective interpretation place someone at an unfair disadvantage when it comes to understanding their social experiences. A situation cannot be explained or understood when the dominant conceptual categories are not known.

Other authors have systematized forms of injustice linked to specific contexts. “Rarefaction of witnessing” [12] is identified as testimonial injustice. This means distorting a testimony by decontextualizing and obstructing it in irrelevant domains [12]. In the field of pain, injustice materializes in the use of strategies to undermine the importance of a sufferer’s situation and its impacts.

Epistemic injustice also exists when an individual or group is excluded from influencing a decision that somehow affects them, despite possessing knowledge that is relevant for that decision. In other cases, injustice occurs when the discriminated individual or group also suffers, at the same time, from other social injustices [13]. For example, patients and doctors who are not recognized as credible lack the influence to establish certain practices as a priority in the operation of the system. This situation has serious consequences such as being potentially deprived of a fair share in collective financial and medical resources [14].

Testimonial injustice occurs in everyday epistemic interactions by contesting the person’s condition as a testifier, either ignoring it, or through personal rejection or insults [15]. This form of injustice can interact with practices of exclusion and oppression [16], too. Likewise, injustice can take the form of willful hermeneutical ignorance when dominantly situated knowers—such as healthcare workers—refuse to acknowledge epistemic tolls developed from the experienced world of those situated marginally, allowing them to misunderstand, misinterpret, and/or ignore whole parts of the sufferer’s world [17].

Referring to pain specifically, epistemic injustice occurs at the individual level of testimony exchange related to pain, but pain is also a situated and relational social phenomenon, so it is necessary to observe other spaces of social relationships. The main social spaces producing systemic injustice that have been analyzed are the labor relations framework, health system, and family- or leisure-related dynamics.

Credibility in the field of healthcare, experts [18], and assessing systems also has to be taken into account because epistemic justice is considered as a virtue of social institutions [19]. Systems require testimonies and evaluations to distribute social resources connected with employment, healthcare, and income security when an individual faces a social risk [20,21].

Individuals are also subject to interpretation as they go through the healthcare system, as the results of body examinations and diagnosis techniques prevail over the patient’s word and descriptions [22]. Particular attention is drawn to the central importance gained by the information provided by diagnostic tests through different technologies in diagnosis, which has ousted the sensations and testimonies offered by individuals to a secondary position, distancing the doctor from the patient [23,24].

Along with the importance that objective tests have gained, there is the distinction between the doctors’ conception of the body (the body as a scientific object) and the sufferers’ (the body in life) [25]. Both factors that occur together put credibility into question when the sufferer’s account is not reflected in the objectified data obtained by diagnostic tests. In fact, the health system has specific tests to detect malingering [26] of a sufferer reporting an organic illness or a mental disorder [27]. This is the case of myalgic encephalomyelitis, back and cervical pain, or contested illnesses [28].

Pain places the sufferer in a “Fragile factuality” because their pain must be “deciphered” by physician [29]. In invisible illnesses, pain can be objectified through biomedical tools [30,31], thus avoiding social stigma. Zajacova [8] analyzes some elements of chronic pain, such as its invisibility, and also of the scientific-medical field, such as some pain being contested. She exposes the complexity that this supposes to face and treat chronic pain.

From a justice perspective, the theory of recognition [32] would also acknowledge the fact that not being recognized involves moral damage to the individual because they may be affected in their dignity and in the achievement of their goals in life [33], and this is empirically shown. In this regard, pain caused by illnesses with a weak institutionalization, as well as pain caused by mental disorders that are lesser-known and have low social legitimacy, will be very degrading for the patient because of their lack of credit. Honneth even called this damage provoked by social order “social pathologies”, but subjects suffer them in the domain of intersubjectivity produced in relation to others [32]. The “chronic pain thus remain an in-between category and patientes regard themselves as liminal, being neither healthy nor legitimately ill” [6].

We think that every pain can cause an epistemic injustice in any area of the sufferer’s life. It is also useful to explain that pain is decontextualized in the sufferer’s lives.

This article attempts to systematize the structural reactions that take the form of epistemic injustice to individuals in (chronic) pain and what consequences this has.

## 2. Materials and Methods

### 2.1. Study Design and Definition of Profiles of Participants

Qualitative in-depth interviews were conducted including inhabitants from different regions of Spain. Most interviews were conducted face-to-face and in person. However, due to the current pandemic and lockdowns, we were faced with the decision of canceling the interviews or continuing using other strategies. We decided to go on, using remote communication tools such as Skype. On two occasions, the interview was conducted over the phone: one of them at the request of the interviewee, the other due to technical problems in the interviewee’s equipment. We dare to say that, even if an in-person interview is the most appropriate procedure, and a more systematic analysis of the results is still pending, we have not noticed significant differences. Some aspects will have to be assessed, such as the participants’ state of mind, eagerness to collaborate because of the topic of the interview, and the exceptional situation. The sample was intentionally selected [34] to include the maximum variability (see Table 1). Each interviewee represents a specific pain. They have been classified, according to its origin, as: chronic pain of physical origin, pain of psychological origin, pain of emotional origin, and pain derived from complex social situations. The International Classification of Diseases (hereinafter ICD-11) and the specialized literature have been followed.

Pain is defined as an unpleasant sensory and emotional experience associated or similar to that associated with actual or potential tissue damage (IASP, 2019). The concept dealt with here is pain with more than three months of evolution [35]. Those that are of physical and psychological origin are coded in ICD-11 as diseases, disorders, or pain. They are biomedical definitions. For the other pains, the concepts used are the following.

Psychological pain is defined in general terms as “a diffuse subjective experience […] associated with major psychiatric disorders” [36]. ICD-11 and DSM V define them as problems in self-functioning, mood disorders, and addictive behaviors that may interfere with personal functions [35].

Emotional pain is a subjective experience that occurs as a response to numerous psychological situations or not. Those that have been produced in response to severe social stressors [36] have been selected here. Social pain is defined as pain suffered when experiencing a negative social situation, such as bullying, isolation, or social exclusion, provoking an emotional response of pain [37].

Two profiles were included in the sample with the goal of tentatively observing two research questions which will be answered in future publications. The chronic fatigue syndrome (in its beginning) was used to compare whether epistemic injustices are the same at the beginning of the pain and when it has already become chronic. It was observed that, in the case of CFS (in its beginnings), the injustices experienced by the sufferer and his perception of them were more related to social aspects than to health aspects.

The profile Comorbidities (MCS. EHS.FM.CFS) was used to observe if the pains were differentiated and attributed to a specific health situation by sufferers. The interviewee can recognize and differentiate corresponding pain to every situation that is mentioned.

Each interviewee was selected for their main pain. The case of comorbidity was selected for having pain originating in the MCS.

The phases of fieldwork, preparing interviews, designing and contrasting scripts, hiring participants, and conducting interviews began in October 2019 and ended in October 2020.

### 2.2. Access to the Field and Sampling Strategy

First, it was decided which pain was going to be included in the sample. To get in touch with the subject who had that profile, we talked and wrote to patient associations. The contact with the people who agreed to be interviewed was achieved through them. Second, a company specializing in fieldwork was hired to find hard-to-find profiles. The company has databases and networks of contacts with other companies throughout the country. The company looks for the person who meets the characteristics required by the research team. Finally, the researchers check the veracity of the information provided and accepts or rejects the candidate.

Table 2 shows the selected pains and their identifiers. The pains in the ICD-11 have an ICD-11 code. The pains that do not appear in this classification are identified by their acronym: pain acronyms.

### 2.3. Data Sources, Instruments, and Data Collection Process

We collected primary data through nineteen in-depth interviews with participants whose profiles are included in Table 2. We used a script of questions applied in a flexible manner, trying to avoid the interrogation effect. The script was structured in three levels: individual (description, evolution and consequences of pain, including stigma, credibility, and subjective experience), structural (family and society consequences), and social policies (healthcare service, diagnosis, and treatment; disability leaves, pensions). Participants were also asked about the difficulties caused by pain and the strategies used to overcome them, as well as the impact of pain on their quality of life.

The first interview was used as a pilot to check the pertinence and thematic depth of the script, the length of the interview, and to identify problems that might arise in the interviewer/interviewee interaction. The average length was 80 min, ranging from 32 to 151 min. Each interview was accompanied by a form including the sociodemographic variables of the participant.

Interviews were carried out in a single stage, but diversity in the types of pain included in the sample and the number of interviews carried out made it possible to achieve saturation of the discourse, that is, “when no new categories or relevant themes are emerging” [38]. In the interview on bullying, no new topics or relevant new categories emerged. Types of injustice and production mechanisms already codified in previous interviews emerged. Specificities of pain emerged as in all interviews.

Research team members checked and provided a critical perspective and assessed decisions made during data analysis [39]. Additionally, a dynamic discussion of quality issues among the research team took place [40].

### 2.4. Data Management and Analysis

Interviews were audio-recorded and verbatim transcribed. The standard transcription guidelines were followed. The interviews were conducted in Spanish. The boxes below provide a translation of the verbatim transcripts.

The type of analysis applied was thematic analysis [41], which includes five stages: (1) familiarization with the texts by successively reading the verbatim transcriptions; (2) identification and codification of data, applying labels to meaning units; (3) application of the code to the body of texts; (4) creation of the data matrix organized into tables or theme groups; and (5) analysis of data organized into broader themes.

We used an inductive–deductive method to obtain the structure of the data, because the analysis of the credibility of pain has been tackled using theoretical concepts from previous research by the same team, and incorporated new codes arising from texts and the categories built in the analysis process. The exposition of the analyzed themes includes quotes from the interviewees, whose identity has been anonymized, and their references to the type of pain have been codified.

### 2.5. Ethics Committee Approval

Each interviewee was thoroughly informed about their participation in the research, orally and in writing, and they signed an informed consent form including all the information about the project and the Ethics Committee approval. The research obtained the Córdoba Research Ethics Committee certificate on 28 January 2020 (file no. 299), stating the complete viability of the research project, considering that its results may be of great interest.

## 3. Results

### 3.1. Initial Findings

Two initial findings are important to stress before going forward with the issue of the sufferer’s credibility. First, pain occurs within the frame of a condition linked to health—be it an illness or not—and it is thus interpreted in relation to such condition. This means that illness, pain, and the consequences of pain are sometimes interchangeable. In addition, pain is a phenomenon situated in space and time and in a social condition and structure defined by social norms, pain care systems, labor relations, and social rights systems. In this study, we use the concept of situated knowledge [42] to refer to the knowledge acquired and expressed by sufferers about their pain, as well as the meaning they give to it, from their particular position. This is why sufferers are not all victims of the same testimonial injustices, nor do they receive them in the same spaces.

Secondly, despite being embodied in an individual, the sensation, perception, and experience of pain are influenced and modulated by situations that take place within the framework of social relations, such as testimonial injustice. Pain has a relational quality which makes a sufferer interpret their pain linked to the social context they belong to [43,44]. Outside of this social context, pain cannot be properly understood by the individual or by those they interact with. Thus, some sufferers express their pain mainly linked to family relationships, to the work place, or to other social experiences, but all of them contribute to pain being experienced the way it is, sufferers being completely aware of the impacts of social relations on their pain.

### 3.2. Spaces Where Epistemic Injustice Is Produced

Disbelief at another person’s pain runs through the entire social structure. When pain is manifest, each social space in which individuals participate sets certain strategies in motion to prevent such pain from having consequences, either on its working dynamics, or on the social reproduction dynamics. Here, we will focus on pain-related epistemic injustice in three social spaces in particular: the workplace, the family, and healthcare settings.

#### 3.2.1. Epistemic Injustice in the Workplace

The rule of the workplace is to keep workers active and productive. When a worker’s pain interferes with the workplace’s dynamics, the work of co-workers, altering their schedules or workload, and when it hinders or keeps the organization from achieving its objectives, the sufferer is discredited using derogatory terms or treated as mentally ill:

“… a woman that is such-and-such years old, middle-aged, what happens to her is that she’s tired or half hysterical, as they say, depressive or something, that’s it, but I’ve never been depressed.”

(About the way she had been treated by physiotherapists.) “physios, they would (…) as if I was half crazy, you know? Yeah, yeah, “go see a psychiatrist”, one said, “I can’t do anything for you, go see a psychiatrist”, because he’d touch me and I’d start crying, but with a tone like (…) you know?”.

(M630.01)

Testimony is also often rarefied [12] by attributing the pain to unrelated problems or situations that take place in other contexts:

[Woman. Migraine is an undervalued pain by her workmates, who are physicians, too. The cause of pain is attributed to something other than the migraine itself.] “They were saying: “but, let’s see, does it hurt so much? Today too? It was a problem for them that I missed work. But if I missed work one day it was because that day was so unbearable that it was impossible. But they didn’t understand it at all. They did not understand it being from the health sector, “this is because of a headache.” No, no, no, this is not a headache, this is such a great intensity, it is such an immense pain that it prevents you from doing any activity because it alters your entire organism”.

(8A80)

Sufferers need to be believed and assessed as responsible and productive workers. Otherwise, their self-esteem and their sense of self would be damaged [15], becoming unreliable for the rest. Being aware of this, these workers may use the following strategies to maintain their credibility.

Occasionally, they make up for the workload lost during a pain crisis, increasing their productivity and taking on work from co-workers:

(Woman. When she is not in pain, she works more than her share in her job to make up for the work she cannot do while she is in pain.) “… but because I think that, when I am ok they see me work so hard, because I try to make up for it, and it’s like I do more, if I can, I take more. If another worker is sick, I do it too”.

(M630.01)

They take medicines to work until pain makes it impossible to do so:

“… calling the neurologist, calling my neurologist, and telling her: I can’t stop working, tell me what I can take (…) And she tells me: “look, tell them to give you an intravenous line, this and that”; delaying consultations for an hour and then keeping on working” (…) Imagine the state I was in. Then, I’d start working even with the catheter on, I would tie the catheter up, because later in the afternoon I had another intravenous dose, I’d tie the catheter up as if I had a splint, as if I had sprained my wrist (…) So that it wouldn’t be seen, and the patients wouldn’t see it”.

(8A80)

They adjust their working hours:

(About job performance. Man. He is able to keep his job because he can organize the amount of work and has some flexibility in working hours.) “… because I’m not at the level of now, of objectives and whatever, you know? So the truth is that I can adapt, I can have that time”.

(SPCFS)

They develop lifestyles that would not interfere with their jobs:

(Woman. Retired prematurely because of her pain. She does not carry out any personal activity in her free time, or leisure, or social relationships. She just rests and sleeps so she can go to work the next day and no one knows her pain. All her activities are aimed at being able to develop the work.) “…you keep going, you keep doing, you keep acting, no matter how much it hurts, no matter how tired you are, if you are exhausted, you just have to go there, work, and your life doesn’t exist”.

(8E49)

They ask for help from other people in the workplace in order to work in the necessary conditions, for instance, in areas free of certain chemicals:

(Woman. Nurse. She asks patients not to wear perfume in her office because the institution does not understand what is happening to her. Her workmates do not believe that the smell of perfume makes her sick.) “I told them I had allergies because they didn’t understand what chemical sensitivity was, that I was allergic to chemicals and, please, don’t wear perfume, and patients were so very nice, I mean, many would do it, they’d keep it in mind”.

(SPCM)

At the workplace as in other social spaces, sufferers must meet conflicting demands: they must be productive to be treated as reliable workers and, at the same time, their behavior and appearance must comply with social representations related to the pain they suffer from. When this is not the case, suspicions about workers’ honesty are often raised or the effects of pain are trivialized to ignore workers’ requests to adjust their working conditions.

In short, it seems obvious that the workplace is an active producer of epistemic injustice.

#### 3.2.2. Epistemic Injustice in the Family

The family is an exceptional environment because of its proximity to the sufferer and the affection-based relationships among its members. The impact of pain on family dynamics produces several types of epistemic injustice. In general, pain is not denied, but the sufferer is expected to have a fairly static or passive behavior. Thus, when pain is manifested through disruptive behaviors or attitudes, the disbelief process is set in motion and any expression or behavior that alters family rules previous to the occurrence of pain calls it into question.

The belief in the truthfulness of pain is built on the exchange of testimonies and on the expression of everyday behaviors related to it. However, family members’ attitudes towards these testimonies and behaviors depend crucially on their understanding of the causes and manifestations of pain, especially before certain types of pain such as bullying, depression, or myalgic encephalomyelitis. Behaviors that are not well understood tend to be assessed—even by close relatives—as avoidable behaviors and character flaws. These behaviors include:

Failing to play the social roles of care and attention—especially in the case of women:

(Mother of a female victim of gender violence blaming her for her situation.)… “you were a fool to leave him”, and my mother didn’t accept my divorce, of course, just like she forced me to get married, she didn’t accept it when I got divorced. “You could be with your children, your husband, your job, and look at yourself now. I’m not going to help you. You asked for it and you are getting worse and you will end up on the streets”.

(SPGV)

Failing to take part in family events:

(Extended family meeting increases the pain of loss, but they insist on the participation of the person who is grieving.) “… I did have some, so to speak, asocial behaviors because, for example, my family, soon after my daughter died, would call me to meet, all together. Sure, all the cousins would be there and all that, except for my daughter. I was like: look, it’s not going to be all of us together”.

(EPLD)

Failing to comply with social customs that are deep-rooted in the family:

(The interviewee feels that the family of his deceased wife does not understand his pain. They behave as if she had not died, inviting him to have fun and participate in parties.) “What am I gonna do at the festival? What am I gonna do at the festival? Who with? “No, you can come with us”, “we’re gonna sing”, “we’re gonna dance”. I’m not dancing without your sister”.

(EPLS)

(Daughters, traditions, and family relations.) “… they have to enjoy the festival, they have to enjoy the Holy Week, whatever they wanna do, what I don’t want is that turning into an imposition from my wife’s family”.

(EPLS)

Failing to comply with social customs related to sexual orientation:

“You know that they loved us, but not like this. In the case of my mother, she said it more than once. She loved me madly, she adored me and protected me so much and until she left us she loved me like crazy, but my mother didn’t want to have a fag son”.

(EPRSO)

Interfering with the celebration of social traditions involving the extended family:

(The interviewee wanted to go to the hospital to be treated but her mother prioritized the Christmas tradition of reuniting with the family.) “This Christmas was terrible, because I’ve been feeling awful and my mother didn’t accept it, because I wanted to go into the hospital to get (…) Not to get well, because my mental health, I don’t want to go to mental health anymore, but at least they adjust your meds, so at least for some days until they adjusted my meds. And my mother didn’t want me to, no way, she didn’t want to, it was Christmas, and it was Christmas, and it was Christmas. What do I care about Christmas? For me Christmas, pardon my expression, was shit”.

(6D10.Z)

#### 3.2.3. Epistemic Injustice in Healthcare Settings

Sufferers attach great importance to epistemic injustice in the field of medical care. Besides the professional’s attitude, there is an institutional dimension of epistemic injustice that transcends the doctor–patient interaction. For instance, hermeneutic injustice occurs when the healthcare system lacks the necessary tools to diagnose and treat pain. This is particularly the case when diseases are still rare and in the process of epistemic construction, recognition, or institutionalization. This situation causes frustration in sufferers, who have feelings of incredulity, lack of framing [45], and discouragement:

“But then I went to my specialists, to the hospital’s internal medicine doctor, and they are very skeptical with the illness, they don’t know it, but it’s true, I have to say, that they say it, I mean: “we don’t know about CFS, it escapes us, we think that we have cases, but it’s not all of this that you’re showing us”. So I was showing very specific tests, and of course they didn’t understand anything at all. And when they saw another test that I got done, I showed the results, they said: “well, come back in 20 years and we’ll see if we are able to interpret them by then”.

(8E49)

“[…] because this has not been well studied yet, both () for family doctors, it’s all Greek”.

(SPCM)

Other cases occur when, from a position of epistemological authority, doctors question their patients’ accounts of pain, calling into question their “somatic culture” [44] to be able to recognize their body signals, areas where they feel pain, or the learning and experience acquired by people with chronic pain:

“I went to the ER and I told the traumatologist: “what’s the matter?”, my back hurts and my seventh vertebra is crushed, and she was like “oh, so what? you come here with a diagnosis already?” She made me get an X-ray and told me: “you are right”. Oh, sure, sure, it wasn’t the first time, when you have that pain in your vertebrae that is so… so specific that you don’t need the doctor, just to get directly… but, of course, you have to go through all the steps”.

(FA0Z)

Testimonial injustice often comes from prejudices in the healthcare field. For instance, when a type of pain with a high prevalence among women is suffered by men, or when the patient’s credibility is put into question for being a woman or an older person:

“… this is a matter of age, but look, I’m 51, not 87. This is menopause, but I’m not menopausal (…) look, you know, no, he was denying what was evident all the time. Sure, if you’re a woman, and of a certain age, well, I’m completely sure that there’s a gender bias, it’s completely clear, completely, it’s like, a woman that is such-and-such years old, middle-aged, what happens to her is that she’s tired or half hysterical, as they say, depressive”.

(M630.01)

The sufferer’s honesty and moral integrity may then be contested by insinuating that they fake their pain to obtain personal, fraudulent benefits:

“… he told me no, there was nothing wrong, I wasn’t sick, I was just making it up to be on sick leave and not go to work”.

(6D10.Z)

### 3.3. Causes of Social Disbelief of Pain

#### 3.3.1. The Subjective Nature of Pain

“There is a unanimous agreement on the idea that pain cannot be vicariously experienced, although it may be intellectually understood and supported from an emotional standpoint. Pains linked to well-known illnesses are more deeply rooted in the social discourse and are better understood intersubjectively. However, there are other pains that are harder to understand even for the sufferers themselves, such as borderline personality disorder “:

(BPD)

“Because she doesn’t know how this feels inside, this is so bad. I’m telling you for real, this is so bad […] she hasn’t understood it yet, and comes here to family gatherings and so on, but hasn’t understood it yet, it’s complicated, right? This is very complicated, because how can you explain to someone that you are hurting inside every day? How can you tell her that you, you, here, that it’s hurting every day? (reaches for heart) How can you explain that? And they have to understand it”.

(6D10.Z)

In addition, the subjective nature of pain makes it difficult to assess both its veracity and intensity by third party observers. However, several circumstances contribute to crediting the sufferer’s testimony and validating their pain. These circumstances include the presence of somatic manifestations that are external (e.g., fractures) or that can be objectivized by diagnostic tests, especially when there are known therapeutic responses, as well as the existence of an underlying illness that is highly socially legitimated (e.g., cancer, diabetes):

“If someone has cancer, then, of course, if they say “I’m in pain”, then they can prove it. It’s a pain that, those pains can be proven”.

(8A80)

By contrast, social disbelief and suspicion of the sufferer’s testimony are fostered by the absence of medical knowledge about the pain’s causes, the absence of effective treatments to relieve it, or the absence of formal recognition by health tribunals:

“… back then I didn’t know yet, I didn’t know anyone who had this and I saw myself as a weirdo, also with complete incomprehension from the people around me. I’m talking about my closest family, such incomprehension that they would say: “those are just your oddities”, “this and that”. This is something that you have to go through. It’s unimaginable”.

(SPCM)

#### 3.3.2. The Social Representations about Pain

The informal knowledge of pain in each context also plays a role in its credibility and it materializes in the form of social representations [46]. These are codes made up of attitudes, perceptions, and norms determining the way women and men act in the world [47]. They make it possible to identify the social factors of perception, thus highlighting the importance of groups and their norms in the representation of social reality [48].

Pain appears in social representations connected to a negative sensation of unease, discomfort, and, on many occasions, suffering. It is also linked to decreased leisure activities, decreased appetite, and untidy appearance reflecting inner malaise. Judgments on credibility are determined by the degree of adjustment between these representations and the behavior of sufferers. When there is no adjustment, suspicion arises about the honesty of the sufferer. Myths and stereotypes play a role, too: for example, “love cures everything” and “pain is forgotten” foster social discredit by diluting the structural causes of pain in adopted children.

Another cause of disbelief is when meaning cannot be found in pain, that is, when its cause cannot be specified, when events happen outside of the social context’s logic, when the consequences of pain are visible, but its causes not quite as much, and when the environment denies or does not accept the existence of the situation. Some cases that correspond to this description are bullying, adoption, and gender-based violence.

Pain interferes with the operation of the social and economic system by hindering the sufferer’s compliance with gender-based social roles, threatening to slow down the profitability of the productive systems, and questioning expert systems, the public administration, and the knowledge system. The credibility of the sufferer thus relies on their trajectory of functional behaviors in the maintenance of social order: compliance with the norms of sexual division of labor, reliable presence and productivity at work, non-interference with group dynamics, and behaviors consistent with the social representations of pain.

### 3.4. Mechanisms Producing Epistemic Injustice

#### 3.4.1. Denying the Causes, Intensity, and Consequences of Pain

The first mechanism is to trivialize the pain of the sufferer by treating it as a minor discomfort, comparing it to the symptoms of common diseases or to other people’s pain in totally different situations, giving advice such as “go for a walk”, “have fun”, or “hang out with friends”, suggesting to grieving parents to make another baby, denying the sufferer the necessary space to manage their pain, pushing to do certain activities, or postponing medical care:

“There are some people who, after 6 years, are still telling me: “don’t complain, the other day my knee hurt a lot too”.

(4A40)

(The mother wants the interviewee to fulfill her roles as a mother by spending more time with her children, even though she cannot do so because of pain.) “You have to spend more time with the kids, you have to spend more time with the kids”. Let me spend two hours feeling good, instead of six and the last four shouting at them. Why do you want that? Why do you want me to be like that? So that in the future their only memory will be my mother shouting?”

(6D10.Z)

Another strategy is appealing to the sufferers’ strength, attributing to them coping abilities superior or inferior to what is considered normal. People can even take themselves as a reference and define as abnormal those behaviors and experiences that do not coincide with their own. Pain, whatever its nature, can also be compared to the loss of a loved one in order to minimize the impact it may cause. Conversely, the sufferer may be invited to compare it to all the elements in their life which are socially attributed a greater importance, such as their children’s wellbeing or their economic security:

“… people before this pain that is a pain that you say, well! This is not a pain from an illness, it’s not a pain that, you know? You can’t justify it as a pain from an illness, or from death, so I think that this pain caused by a breakup is trivialized: “well, you have the kids, everything is fine, you have a job”.

(EPB)

#### 3.4.2. Distorting and Rarefying Testimonies of Pain

A second mechanism is to distort and rarefy pain testimonies by altering the intention or reasons of those expressing pain, insinuating that their complaints are a demand for attention [12]. For example, when behaviors aimed at reducing pain are deemed unconventional or problematic (e.g., self-harm), they are not accepted as an attempt to ease pain, just like the sufferers express it, but as a self-interested action to call for more attention:

“… when I’ve had very nasty moments, like cutting myself and so on, and not many people know it because it shocks people a lot and so I’m a little more careful, eh, so that’s it, a call for attention […] I tried to kill myself in the past, see? Some years ago, that’s why I was hospitalized, and then, back then, it was also a call for attention”.

(PsPCD)

Credibility is also distorted when common events in any person’s life are attributed to pain, thus negating their complexity as a human being and their capacity to have social relations on the basis of their own criteria.

#### 3.4.3. Individualization of Pain

A third mechanism is the individualization of pain [49]. People may see pain as exclusively dependent on each sufferer’s actions, on their deeds, setting aside the analysis of its structural causes and social determinants [9]. This occurs when pain is caused by violence or associated with illnesses such as addictions, which are still often assessed as will-dependent behaviors. People may also blame the sufferer by holding them responsible for their pain, taking for granted that they sought it out or that they could have avoided it. A different frequent strategy is to make the sufferer feel embarrassment when pain interferes with their everyday activities, such as doing their job or not being able to play the assigned social roles, particularly in the case of pains which are not widely socially legitimized yet.

Blaming the individual overlooks the social factors affecting them, such as their position in the social structure, living conditions, being a victim of violence, or vulnerability trajectory. It also ignores the sufferers’ subjectivity and the possible psychological dispositions leading to painful situations. All in all, the attribution of blame is not a very elaborate and rather abstract reaction, although it causes additional pain.

Systematic suspicion occurs when any behavior on the part of the sufferer is judged and described as a sign of their lack of honesty. Obesity is a paradigmatic case of this strategy, as the sufferer will be watched in all their food-related behaviors and they will be interpreted as suspected of simulation.

“If we are eating something and we are fat, they say: “look how much he is eating and he does not know that he is getting fat” (…) And if you are eating lettuce, they will say: that one is there eating a little lettuce, but that one will fill herself up at home. He is pretending everything there. So, we are always questioned. They are always looking at you. We are always doing the wrong thing”.

(5B81)

Systematic suspicion also occurs when a lesser-known pain (e.g., multiple chemical sensitivity) appears in two members of the same family.

Another individualization strategy is questioning the sufferer’s psychological balance, because from then on, their declarations about pain can be questioned. Although each type of pain generates its specific label, “madness” calls into question all and any testimony, because it negates the sufferer in their most essential nature. This leads the sufferer to silence for fear of being considered eccentric, unreliable, and excluded from the system:

(At school she was told) “look at her, she’s as nuts as her granddaughter”.

(SPB)

“[…] there, at the healthcare center, everyone looked at her as “the nut”. And this doctor told my sister: “oh, my, I feel so bad for thinking that that girl who came with a facemask on was crazy. Me and other colleagues, we thought that she was mad”.

(SPCM)

### 3.5. Impact of Epistemic Injustice

Disbelief increases pain and causes emotional suffering. Sufferers report that, when their testimony was put into question or when their personal credibility was eroded, they felt great moral suffering. This is particularly the case with chronic pain. Conversely, when a sufferer feels understood and well-treated, credibility acts as a therapeutic element, improving and easing pain. On the one hand, disbelief leads to self-censorship when sufferers are aware that talking about their pain overwhelms those listening or demands too much attention. In this case, they limit their expressions of pain to avoid losing their legitimacy as sufferers. Additionally, when pain is misunderstood or ignored by others, the sufferer internalizes that nothing can change in their reality, which leads to feelings of learned helplessness. However, talking about pain has beneficial effects, as it increases self-legitimacy and meaning, locating pain at the level of human experience. On the other hand, the internalization of the disbelief and suspicions of dishonesty in their social environment may lead some sufferers to self-stigma, for example, a temporary disability leave being internalized as a dishonest behavior.

When pain involves a shift in the sufferer’s life and activities, this may result in isolation and expulsion dynamics. Each type of pain causes a different dynamic, although there are common features. When pain comes from child loss, the environment disappears because they are unable to face that pain. When pain is caused by obesity, sufferers who are unable to follow the group dynamics are excluded from its activities. In other cases, isolation and exclusion occur to avoid being labeled or because pain is disruptive and incomprehensible.

The sufferer whose behaviors are not understood as direct manifestations of pain may also be infantilized. This is frequent when a family member takes care of the sufferer. Care norms are then mixed with other norms, such as controlling their relationships, deciding when to go to the doctor, or assuming they lack judgment. This is particularly the case in pains coming from psychological and social problems.

Epistemic injustice creates barriers to accessing the public healthcare system and appropriate treatments for pain. This is especially the case when a type of pain or its causes are not well understood, either because the system lacks the resources to conduct research on it or because it lacks resources to provide adequate care, as it does for other patients. This also stems from the prejudices of professionals who, given the lack of an analysis framework, are systematically suspicious of the sufferer’s testimony.

These same barriers are encountered when seeking legal recognition of the sufferer’s situation. They produce a constant feeling of unfair treatment with respect to other people who suffer as well as deterioration of their living conditions.

Epistemic injustice has material consequences for victims in the workplace, too. When the organization does not acknowledge or accept the limitations that pain can cause, it may deny or ignore the worker’s requests to adjust their working conditions or for mobility within the organization. In such a case, there is no alternative but to quit the job.

**Figure 1:**
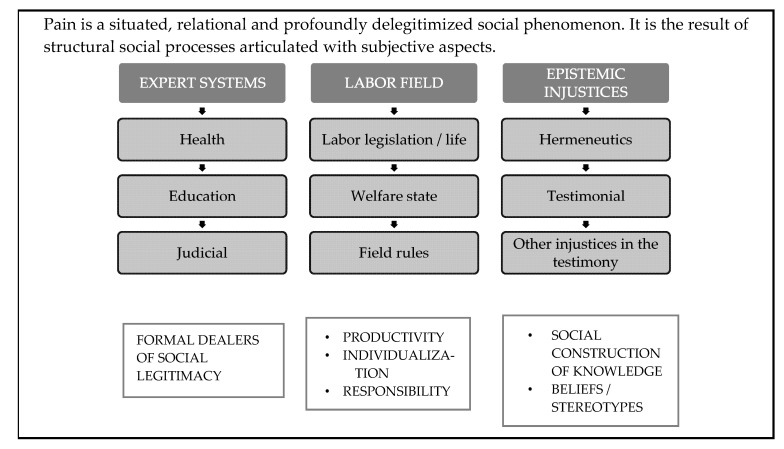
Analysis of the results. Source: Prepared by the authors.

A schematic analysis of the results can be seen in Figure 1.

## 4. Discussion

There are different forms of epistemic injustice, including but not limited to testimonial and hermeneutic injustices. Testimonial injustice is the result of a prejudice taking place in the framework of a relation of power, while hermeneutic injustice takes place when a situation cannot be explained or understood using the dominant conceptual categories [11].

People in pain are victims of these injustices due to prejudices and stereotypes. They also suffer a secondary victimization because, when they are cared for, the concept of pain proposed by the biopsychosocial model is not applied exhaustively. In addition, painful ills are contested in the field of medicine [30] and in social contexts, too, as corroborated by results.

The credit given to the testimony of a sufferer is the result of a judgment that depends on each of the systems that the individual goes through. They take into account three items to evaluate the sufferer’s credibility: his historical veracity, what he says about pain, and the way he says it. All items must be consistent with the listener’s knowledge about the sufferer.

Through this formal definition of credibility, the social order maintains its stability and protects the legal certainty of individuals, defending them from those who intend to take advantage of the system, protecting them against malingering. However, by doing so, the social order also causes other kinds of epistemic injustice to other individuals who are wrongfully and unjustifiably discriminated against as knowers.

Results show how the different types of injustice that have been conceptualized occur in everyday life, including the case of sufferers of contested diseases.

In fact, the forms of injustice that Fricker deals with may not gather all the situations that have been empirically observed in the specific experience of pain, nor the number of nuances with which injustice is perceived.

“Rarefaction of witnessing” [12] is identified as testimonial injustice, which means distorting a testimony by decontextualizing and obstructing it in irrelevant domains [12]. In the field of pain, it is one of the mechanisms causing disbelief of the sufferer’s testimony in all the domains of social life, where pain is decontextualized and minimized, or compared to situations lived by any other person, turning it into a different phenomenon from that expressed by sufferers. These reactions put them in a position of complete incomprehension and isolation.

One of the questions that we often ask ourselves in research about the social perception of pain is whether we are evaluating pain itself or the sufferer when we evaluate the meaning of pain. We think we have found a satisfactory answer in the analysis. We observed that, apart from the injustices due the testimony to prejudices [11], other injustices occur during communication. They will take the form of ignoring or rejecting the individual [15] sufferer who is reporting their pain as such, either because they are attributed obscure interests or because they are considered to be indolent or responsible for their situation. This is usually so in the case of known people whose pain is framed within their decisions and activities, for example, obesity.

The probability that an individual will suffer an epistemic injustice in the act of expressing their pain is high, regardless of the type of pain. All the interviewees expressed concern and unease because they were not considered to be sufficiently believable or reliable.

In the workplace, credibility is linked to the worker’s reliability to fulfil their responsibilities and not to be assessed as a cheat who appropriates their workmates and employer’s time and effort. In the family and leisure domains, it is mainly linked to playing the roles socially assigned on the grounds of the sex–gender system and to giving continuity to group dynamics.

Some medical professionals believe that it is necessary for scientific evidence not to serve to separate doctors and patients, but to bring them closer [50]. Hutchinson and Rogers [51] propose several elements that might improve clinical decisions, namely, using more heterogeneous methods that increase the reliability of knowledge and hermeneutical tools in the interpretation of data (quoting Fricker, in particular), and admitting that experience, intuition, and tacit knowledge are reaffirmed as reliable contributions to knowledge in a doctor’s office. When a sufferer’s voice is canceled in favor of diagnostic tests and techniques, they are placed in a situation of epistemic vulnerability [24], because their knowledge and testimony are not accepted as reliable evidence of their suffering.

This situation occurs in cases such as multiple chemical sensitivity or bullying, with an important social component that the health system is unable to measure or to provide care for patients at its levels of care. However, it is important to address the complaints of these kinds of sufferers, because their demands on the healthcare system do not only seek pain management, but also recognition of the legitimacy of its causes, and of themselves as sufferers. This situation could be included among those that Honkasalo [6] names as “vicissitudes of pain and suffering: Chronic pain and liminality”. The search for legitimacy is constant among people with chronic pain.

Other cases reveal that sufferer’s testimony is ignored when pain is not dealt with with the specificity it would require. This standardization comes from practices such as providing a list of causes and general recommendations for pain or signs that do not respond to the situation brought up by the sufferer at medical practices.

Patients suffering pain do not perceive diagnostic tests as an obstacle. They believe that it would be necessary to advance the technical and scientific knowledge to know and understand their pain better and to modify aspects of the organization that produces different kinds of injustices for every sufferer.

“Expert patients” [24] have also taken part in this research. These are patients suffering chronic pain who know the state of scientific knowledge and know the way the administration and the healthcare system function well, and they are able to interpret their pain experiences using medical categories. These patients would be expected to be treated with a higher degree of epistemic justice than the rest. However, this is not the case. Their testimonies are not useful enough to provide a diagnosis, nor does the current state of knowledge accept individual testimonies as a category under which to interpret the reality of pain.

## 5. Conclusions

As pain is being researched, certain aspects of its nature are revealed, which qualify it as a phenomenon of enormous complexity, which requires a transdisciplinary approach in order to comprehend and analyze it better. From its initial conceptualizations in the field of medicine, some aspects of its nature have been discovered, having to do with human psychology, and also with the social structure where individuals and social groups interact.

Pain is not a neutral phenomenon, nor is it a priori socially accepted, but the society where it occurs and the contexts where sufferers interact are of utmost importance in its definition and virtuality.

When pain occurs, the affected person is going to observe that all the areas of their life are going to react to it. In the workplace, pain undermines the foundations of its definition and organization. Everything that takes place in the workplace aims to avoid the impacts of pain on production and to preserve the organization’s dynamics, preventing employees from stopping working. In family relations, the reaction intends to keep the reproductive functions intact, and, with them, the development of the tasks and behaviors of the social roles attributed on the grounds of sex, as well as to give continuity to this institution, maintaining the celebration of traditions and family events. Among friends, the reaction is to prevent group dynamics from being altered by pain, by the behaviors associated with it, or by the prescribed medical treatment, with the aim of maintaining the group’s usual activities. Finally, expert systems, such as the healthcare system, the social security system, and the knowledge system will try to prevent pain from altering the organization, the professional practice, the evaluation and certification systems, and the diagnostic devices. However, pain, as defined by the International Association for Pain Study, is “an unpleasant sensory and emotional experience associated with, or resembling that associated with, actual or potential tissue damage”. Such concept assumes that many people suffer pain without having suffered damage, or without known pathophysiological causes, but its experience cannot be distinguished from the former. Therefore, it is understood that if a patient regards their experience as pain, it should be accepted as pain (IAPS), and as knowledge about pain advances from a multidisciplinary perspective, it is an important challenge for those systems.

In situations of social life related to disability and mental illness, the steps taken by the public administration in the exercise of its powers or the exclusion of communication between adults are examples of how testimonial injustice, based on prejudices and in stereotypes [11], produces clear situations of social exclusion.

Herzog (2021) [52] understands that there are collective evaluations that expel from society those who cannot express themselves using the accepted modes of expression. In this sense, social exclusion is a structural phenomenon because it is part of social functioning, which acts by establishing differences between subjects who deserve equal treatment in their capacities as agents of knowledge. This situation has impacts on healthcare, communication, intellectual self-trust [53], on the life and death of citizens [54], and on democracy.

We observe that, before pain, social order reacts by trying to preserve its norms and internal functioning dynamics, using different strategies, one of them being epistemic injustice [11,12,15,55], which will take different forms depending on the context where the communicative interaction takes place.

## Figures and Tables

**Table 1:** Sociodemographic characteristics of interviewees.

Sociodemographic Characteristics *N* = 19		Physical Pain *N* = 6 (31.6%)	Psychological Pain *N* = 4 (21.1%)	Emotional Pain *N* = 4 (21.1%)	Pain of a Complex Social Origin *N* = 5 (26.3%)
Age	Average, (range), years	55 (29–84)	41.8 (35–50)	52.5 (44–56)	48.2 (42–56)
No. of children	Average, (range)	1.3 (1–4)	2 (0–2)	1.5 (0.2)	2 (2)
Sex	Man	1	2	3	1
	Woman	5	2	1	4
Education	Primary education	1	-	-	-
	Secondary education	-	1	-	-
	Vocational training	1	1	-	1
	University	4	2	4	4
Marital status	Single	2	2	1	-
	Married/has a partner	2	-	1	4
	Divorced/separated	-	2	1	1
	Widowed	1	-	1	-
Habitat size	<20,000	-	-	-	1
	20,001–100,000	2	1	1	-
	>100,001	4	3	3	4
Employment	Employed	3	3	4	4
	Unemployed	-	1	-	1
	Retired/Disability leave	2	-	-	-
	Housework	1	-	-	-
Occupation	Qualified	4	2	4	4
	Unqualified	2	2	-	1
Participates in association	Yes	4	4	2	2
	No	2	2	2	3
Total Age	Average, (range), years	49.89 (29–84)			
Total no. of children	Average, (range)	1.37 (0–4)			

Source: Prepared by the authors. Sociodemographic characteristics of the sample’s participants.

**Table 2:** Sample. Origin of the pain. Pain. Identifiers of pain.

Origin of the Pain	Pain	Identifiers of Pain
		ICD-11 CODES *
PHYSICAL	Osteoarthritis	FA0Z
	Chronic migraine	8A80
	Obesity	5B81
	Generalized pain (fibromyalgia)	M630.01
	Lupus	4A40
	Post-viral fatigue syndrome (myalgic encephalomyelitis)	8E49
PSYCHIATRIC/ PSYCHOLOGICAL	Schizophrenia (schizoaffective disorder)	6A20
	Borderline personality disorder	6D10.Z
	Chronic depression	MB245
	Gambling addiction	6C50.Z
		ACRONYMS **
EMOTIONAL IMPACT	Daughter’s death	EIDD
	Wife’s death	EIDW
	Relationship breakdown	EIRB
	Rejection due to sexual orientation	EIRSO
COMPLEX SOCIAL SITUATIONS	Chronic fatigue syndrome (in its beginning)	CSSCFS
	Comorbidities. MCS. EHS FM CFS	CSSCM
	Gender-based violence	CSSGBV
	Bullying	CSSB
	Owing to adoption	CSSA

Source: Compiled by the author; * Pain listed in the ICD-11 classification. Henceforth referred to by their ICD-11 code.; ** Pain not classified in the ICD. Henceforth referred to by their acronym.

## Data Availability

Data is unavailable due to privacy or ethical restrictions. The complete transcripts of all the interviews carried out in the investigation and described in the material and methods section cannot be made public in any type of repository, because they could identify the people interviewed in breach of the anonymity and privacy commitment that we have with them.
